# Previously Unreported Case of Variant Posterior Tibial Vein Forming a Loop Adjacent to the Posterior Tibial Artery

**DOI:** 10.7759/cureus.22248

**Published:** 2022-02-15

**Authors:** Preston M Terle, Joe Iwanaga, Łukasz Olewnik, R. Shane Tubbs

**Affiliations:** 1 Department of Structural and Cellular Biology, Tulane University School of Medicine, New Orleans, USA; 2 Department of Neurosurgery, Tulane University School of Medicine, New Orleans, USA; 3 Department of Anatomical Dissection and Donation, Medical University of Lodz, Lodz, POL; 4 Anatomical Sciences, St. George's University, St. George's, GRD; 5 Neurosurgery and Ochsner Neuroscience Institute, Ochsner Health System, New Orleans, USA

**Keywords:** cadaver, anatomy, tibial nerve, posterior tibial artery, posterior tibial vein

## Abstract

The posterior tibial vein (PTV) is formed distally by the medial and lateral plantar veins and ends proximally at the joining with the peroneal vein. Variations of the PTV can result in unique clinical presentations. Such variations at the proximal location have been classified previously, but few have been identified distally. In an adult male cadaver, we identified a unilateral distal PTV variation that bifurcated posterior to the medial malleolus. This bifurcation rejoined inferiorly to the medial malleolus and formed a loop that was transected by the posterior tibial artery from deep to superficial. Although this PTV variation is rare, we believe it could be clinically significant for tarsal tunnel syndrome (TTS) and catheter-directed thrombolysis (CDT) of deep vein thrombosis (DVT). Such anatomical variations should be documented and added to clinical databases to improve patient outcomes and diagnostic techniques.

## Introduction

In normal anatomy, the distal end of the posterior tibial vein (PTV) is formed by the merger of the medial and lateral plantar veins. Thereafter, the PTV runs posterior to the medial malleolus, between the posterior tibial artery and the tibial nerve. The PTV and the peroneal vein combine to form the tibioperoneal trunk, which is later united with the anterior tibial vein to form the popliteal vein. These veins form a portion of the peripheral venous system, specifically the subfascial system, which is interconnected with the epifascial system via perforating veins. The PTV thereby drains the plantar aspect of the foot, the ankle, and the posterior leg [1,2]. Anatomical variations in it can affect patient outcomes in a variety of procedures and also constitute unique causes for clinical conditions. In this case report, we specifically highlight tarsal tunnel syndrome (TTS) and catheter-directed thrombolysis (CDT). Some variations increase the surface area of the PTV, thereby potentially increasing the risk of TTS owing to the greater likelihood of a PTV aneurysm [3]. We also believe that the variation in the PTV described here could increase the risk of thrombosis formation and potentially make subsequent treatment through CDT more difficult [1,4]. Drawing attention to anatomical variations in the PTV can help foot and ankle specialists in treating tarsal tunnel syndrome and vascular surgeons in treating deep vein thrombosis (DVT).

## Case presentation

**Figure 1:**
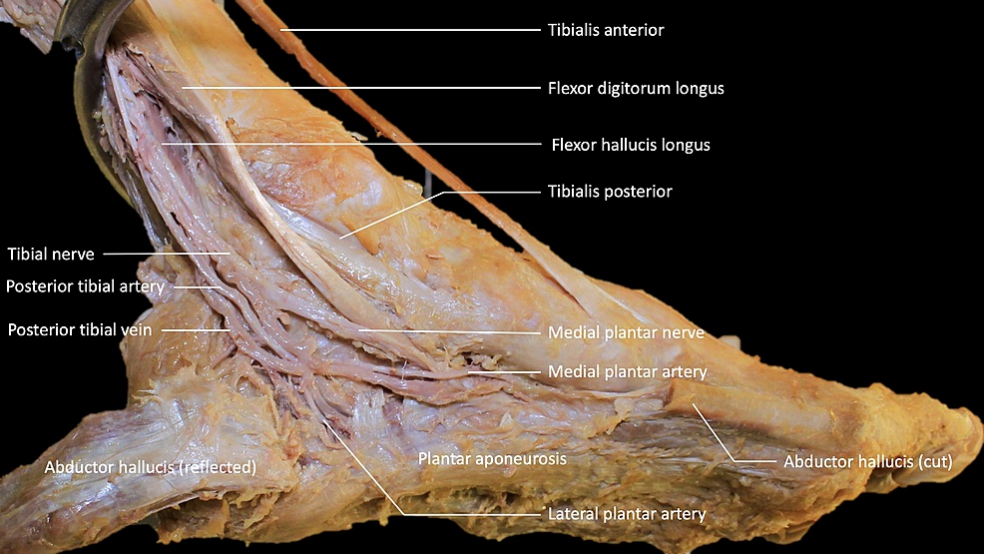
Variant posterior tibial vein found at the posterior malleolus on the left foot.

During routine dissection of a male cadaver aged 71 at death, a variant PTV was found in the left ankle (Figure 1).

**Figure 2:**
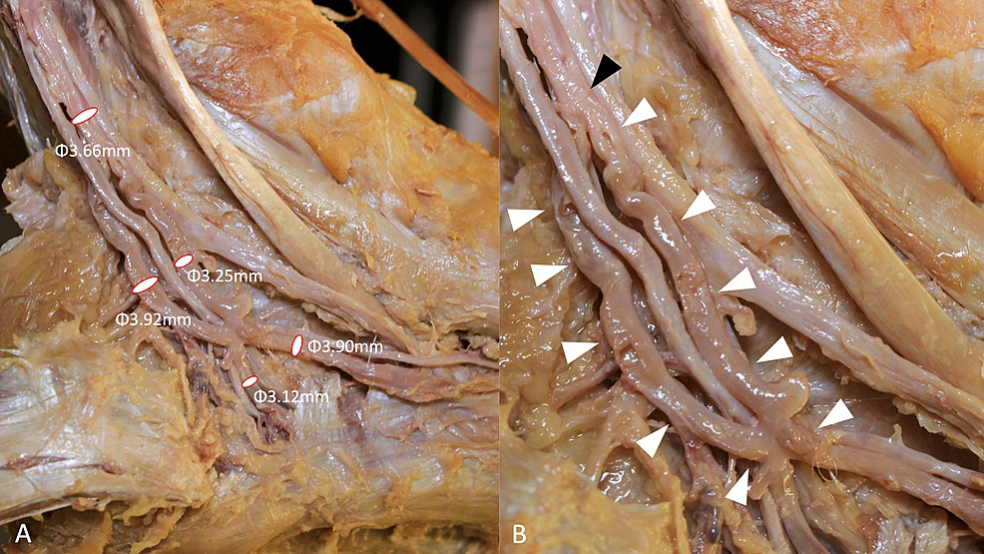
Magnified views of the variant posterior tibial vein. (A) Diameters of the tributaries of the posterior tibial vein are shown and (B) magnified view of image (A). Note that a loop (white arrowhead) formed by two tributaries joins to form a posterior tibial vein (black arrowhead).

The two veins running parallel to the medial and lateral plantar arteries joined lateral to the posterior tibial artery and posterior malleolus, then bifurcated immediately into two veins. Both ran posterosuperiorly parallel to the PTV and merged to form one PTV medial to the posterior tibial artery (Figure 2).

This confluence of two veins medial to the posterior tibial artery formed a loop; the posterior tibial artery traveled through this loop from medial to lateral. Other parts of the foot and ankle joints were normal. No venous variation was found in the right ankle. There were no obvious surgical scars in the area dissected.

## Discussion

Embryologically, the first bud of the lower limbs appears a few days after the upper limbs. At week 5, the primitive anterior tibial vein appears. By week 7 to 8, both anterior and posterior tibial veins are seen. Thus, the variant vein discussed in the present report is considered to have formed by then. PTV variants have been classified previously by their locations distal to the beginning of the popliteal vein, specifically where the PTV and fibular vein merge at their respective ends [1]. The variation presented in this case study was formed more distally than previous studies have described, lateral to the medial malleolus. We believe that this anatomical variation is important for two clinical scenarios: the formation of thrombosis and the increased risk of developing tarsal tunnel syndrome. During ankle fractures and surgery, the PTV was the second most likely vein to form a DVT with a frequency of 18% [5]. Understanding the potential for variations in the PTV could be important for determining patient risk during surgery and the accompanying risk of DVT [4]. Previous findings have also shown an increased potential for thrombosis with variations in the PTV, in general, owing to greater collateral formation [6]. Such thrombi contribute to DVT and, thereafter, potentially to pulmonary embolism. Treatment for DVT is catheter-based thrombolysis (CDT), which is used to dissolve thrombi and preclude embolism formation. The PTV can be used for CDT, but there are conflicting opinions about its efficacy because of the number of possible variations, especially in the distal segments, in contrast to the anterior tibial vein, which has a larger surface area and less variation [2].

TTS generally results from impingement on the posterior tibial nerve. One potential cause is a PTV aneurysm impinging on the posterior tibial nerve. We believe that this case presentation, revealing an anatomical variation in the distal PTV, could entail an increased likelihood of TTS because the extra branch of the PTV within the tarsal tunnel decreases the unoccupied volume of the tunnel itself. The increased surface area of the distal PTV could also increase the risk of a PTV aneurysm. Both factors could contribute to TTS via impingement on the posterior tibial nerve. The tibial nerve branches into the medial and lateral plantar nerves and the calcaneal nerve within the tarsal tunnel and runs between the tendons of the flexor digitorum longus and flexor hallucis longus muscles. In less than 5% of people, this occurs prior to the tarsal tunnel. TTS can be caused by multiple extrinsic or intrinsic factors, including inflammation, scarring, anatomical variation, ischemia, or deformity of the posterior tibial nerve located deep in the flexor retinaculum [3,7]. The flexor retinaculum is formed by the deep and superficial aponeuroses of the foot [3,8]. The clinical presentation of TTS varies widely, making its diagnosis difficult; symptoms include burning, numbness, or paresthesia over the plantar aspect of the foot and/or the medial aspect of the ankle. Localized pain over the flexor retinaculum itself is the predominant diagnostic symptom [9,10]. Surgical release is used to treat TTS when conservative methods fail, obviating 85-90% of prior symptoms (Abstract: Reade BM, Longo DC, Keller MC. Tarsal Tunnel Syndrome. Clin Podiatr Med Surg; 2001 Jul;18(3):395-408). TTS is poorly understood and can therefore lead to highly variable outcomes, which can, in turn, lead to recurrent issues owing to the incomplete release of the nerve entrapment, variation in the nerves, injury to the contents of the tarsal tunnel, and subsequent scarring, all contributing to a recurrence rate of 5% to as high as 40% [11-13].

## Conclusions

The PTV variant presented in this case report can contribute to injuring the contents of the tarsal tunnel if a surgeon is unaware of it, therefore potentially increasing the risk of recurrent TTS. We believe that further studies should be conducted to discover other anatomical variations in the PTV, leading to a greater clinical understanding of the PTV and in turn improving the outcomes of CDT treatment for DVT and TTS release.
